# Development of Fiber-Bragg-Grating-Integrated Artificial Embedded Tendon for Multifunctional Assessment of Temperature, Strain, and Curvature

**DOI:** 10.3390/s23177332

**Published:** 2023-08-22

**Authors:** Robertson Pires-Junior, Anselmo Frizera, Carlos Marques, Arnaldo Leal-Junior

**Affiliations:** 1 Graduate Program in Electrical Engineering, Federal University of Espírito Santo, Vitoria 29075-910, Brazil; robertson.pires@edu.ufes.br (R.P.-J.); frizera@ieee.org (A.F.); 2 Department of Physics and I3N, University of Aveiro, Campus Universitário de Santiago, 3810-193 Aveiro, Portugal; carlos.marques@ua.pt

**Keywords:** multifunctional structures, artificial tendon, FBG sensors

## Abstract

This paper presents the development and application of an optical fiber-embedded tendon based on biomimetic multifunctional structures. The tendon was fabricated using a thermocure resin (polyurethane) and the three optical fibers with one fiber Bragg grating (FBG) inscribed in each fiber. The first step in the FBG-integrated artificial tendon analysis is the mechanical properties assessment through stress–strain curves, which indicated the customization of the proposed device, since it is possible to tailor the Young’s modulus and strain limit of the tendon as a function of the integrated optical fibers, where the coated and uncoated fibers lead to differences in both parameters, i.e., strain limits and Young’s modulus. Then, the artificial tendon integrated with FBG sensors undergoes three types of characterization, which assesses the influence of temperature, single-axis strain, and curvature. Results show similarities in the temperature responses in all analyzed FBGs, where the variations are related to the heterogeneity on the polyurethane matrix distribution. In contrast, the FBGs embedded in the tendon presented a reduction in the strain sensitivity when compared with the bare FBGs (i.e., without the integration in the artificial tendon). Such results demonstrated a reduction in the sensitivity as high as 77% when compared with the bare FBGs, which is related to strain field distributions in the FBGs when embedded in the tendon. In addition, the curvature tests indicated variations in both optical power and wavelength shift, where both parameters are used on the angle estimation using the proposed multifunctional artificial tendon. To that extent, root mean squared error of around 3.25° is obtained when both spectral features are considered. Therefore, the proposed approach indicates a suitable method for the development of smart structures in which the multifunctional capability of the device leads to the possibility of using not only as a structural element in tendon-driven actuators and devices, but also as a sensor element for the different structures.

## 1. Introduction

The development of multifunctional structures has been motivated due to the advances and wide spread of different sensor systems that can be embedded in various structures [1]. In addition, the demands of smart devices and structures commonly discussed in the internet of things context resulted in the development of different flexible sensor systems with the capability of embedment in complex structures [2]. For this reason, the efforts in multifunctional structure development find important applications in structural health monitoring [3], robotics [4], industrial process monitoring [5], and biomechanical applications [6], where the latter is directly related to the wide spread of assistive tools, bio-engineering, as well as biomimetic devices [7].

In this context, the understanding of the chemical, physical, and biological concepts presented in nature can lead humanity to develop technologies capable of exceeding the fundamental performance and the functional limitations of materials [8]. Biomimetics is the application of these natural concepts by imitating biological structures and compositions, which among the areas of human knowledge have wide applicability in the development of sensors. That said, one of the areas of study that use the biomimetic approach is aimed at developing embedded sensors from multifunctional materials. In addition, among the geometries and functionalities present in nature, there is particular interest in the study and development of artificial tendons. Tendons are present in humans with the function of connecting bone to muscle and are capable of withstanding traction; however, they can partially or completely break. Therefore, to replace a ruptured tendon, it is possible to resort to an artificial prosthetic tendon, which must be made of biocompatible material. Furthermore, the artificial tendon is used in orthosis for the rehabilitation of members of people affected by stroke [9].

As a common approach in the development of multifunctional structures, the sensor integration can offer not only the structural functionality but also the capability of sensing different parameters in the environment as well as in the structure itself [10]. To that extent, the optical fiber sensors present intrinsic advantages related to their small dimensions and weight that make them suitable for the integration in different structures [11]. In addition, the chemical stability and electromagnetic field immunity enable their application in harsh environments and in conjunction with electrical motors and components [12]. Another crucial advantage of optical fiber sensors is their multiplexing capabilities in which different sensors can be inscribed in a single optical fiber [13]. Thus, the optical fiber sensors are embedded in different materials through approaches such as micromachining [14], welding [15], additive manufacturing [16], and thermocure resin integration.

Considering the advantages of optical fiber integration in different structures, there is a multitude of optical fiber sensor approaches conventionally employed and proposed in the literature [17]. Among these different approaches, fiber Bragg gratings (FBGs) are one of the most commonly used optical fiber sensor approaches, where such wide spread occurs due to the advantages of easy fabrication and signal processing in conjunction with the direct multiplexing capability through the wavelength division multiplexing [13]. For this reason, the use of FBG sensors and their variants has been proposed for different applications from structural health monitoring [18] to biomedical applications using multifunctional structures [19]. It is also worth noting that there are many technologies for temperature, curvature, and strain sensing. If the conventional electromechanic sensors are considered, the sensors generally suffer from electromagnetic field sensitivity (an important drawback in actuation systems) and there is an issue on sensor embedment in flexible structures [20]. In addition, flexible sensors have been proposed, which generally present high hysteresis and harm the multifunctional structure accuracy. To that extent, the optical fiber sensors are preferable in such specific applications, especially the FBGs that also include the multiplexing capabilities and multiparameter sensing possibilities.

The development of optical-fiber-embedded smart structures includes orthosis [20], integrated concrete, and civil structures [21] as well as smart textiles [22] and integrated mechanical components, such as diaphragms [23], cantilevers [24], and flexible hinges [25]. If the biomimetic smart structures are considered, the development of optical fiber sensors for tendon instrumentation can lead to important advantages in the design and control of soft robotics devices [26]. Such instrumentation approaches lead to another step forward towards a fully integrated sensor system, where the development of an optical-fiber-integrated smart tendon was proposed in [27]. In this case, the intensity variation optical fiber sensor approach is used to obtain a sensor system for continuous strain monitoring in an artificial tendon [27]. However, the use of FBGs integrated in artificial tendons can lead to additional data on the optical-fiber-integrated artificial tendon, since the multiplexing capabilities of FBG sensors result in the possibility of developing a multiparameter sensor system as well as the shape reconstruction of the tendon device due to the possibility of measuring the strain distribution along the artificial tendon. Considering this background, this paper presents the development of an artificial tendon with integrated optical fibers in different positions. In addition, each fiber has an FBG inscribed that leads to the possibility of simultaneous assessment of different parameters such as temperature, strain, and curvature as well as the strain distribution along the tendon due to the differences in the position of the FBGs along the proposed artificial tendon. The novelty of the proposed design is the combination of multifunctional and multiparameter capabilities of the artificial tendon designed, where there is the embedment of FBGs into the artificial tendon structure to enable the multifunctional characteristics of the proposed device, which not only provides structural capability of the sensor system but also the self-sensing aspects. This novel design provide new paradigms in actuation systems in which self-sensing structures can provide additional information for controllers. In addition, the multiparameter sensing capabilities enable the measurement of different conditions in the tendon and in the structure at which the tendon is attached.

## 2. Materials and Methods

The use of optical fibers as sensors of physical parameters, such as temperature, deformation, and bending, is already widespread in the literature, mainly with the application of fibers whose core is engraved with Bragg gratings (FBGs). FBGs are inscribed into the optical fiber core when exposed to ultraviolet (UV) radiation emitted by an external source [28]. The UV beam, when crossing the phase mask, is divided into periodic beams, which are concentrated in their respective focal points positioned in the fiber core. UV radiation causes a localized refractive index change. As the core is hit with several beams, a mirror net is formed. The periodicity between the mirrors will determine which wavelength will be reflected when exposed to a wide band of wavelengths. The FBGs are sensitive to the direct variation of these mentioned parameters, as can be seen in the following equation that was deduced in [29]:

(1) $$\Delta \lambda_{B}=\left[\left(1-P_{e}\right)\Delta \varepsilon +\left(\alpha +\zeta \right)\Delta T\right]\lambda_{B}$$

where Δε represents the strain variation and ΔT is the temperature variation. In addition, optical fibers possess several advantageous characteristics that make them well suited for sensing applications. These include their immunity to electromagnetic fields, compactness, multiplexing capabilities, and chemical stability. Among these advantages, their chemical stability is particularly noteworthy as it enables the integration of optical fibers into various materials.

**Figure 1:**
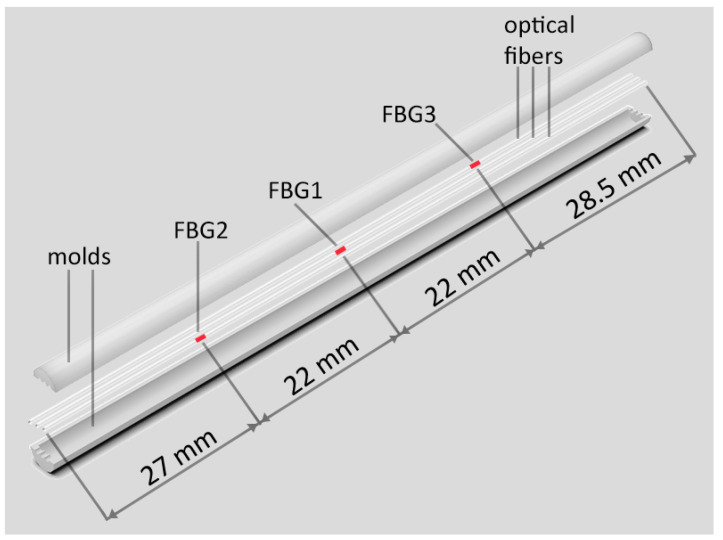
Setup used for tendon fabrication.

As silica optical fibers have dimensions on the micrometer scale, their structures are fragile, so they require protection layers to avoid direct impact. One of the functions of integrating optical fibers is to overcome their fragility. Another purpose of integration is to provide a measurement of a desired parameter. This measurement will have affected sensitivity depending on the structure and positioning of the optical fiber in it. In this case, the use of optical fibers in conjunction with the flexible resins can lead to a customization of the artificial tendon mechanical properties, which is motivated by the biomimetic design of a tendon. In this case, there is a multimaterial design in which the tendon has a composite structure with a matrix of flexible material (i.e., smaller Young’s modulus), whereas fibers with higher Young’s modulus are integrated in this matrix as schematically depicted in Figure 1. To that extent, the tendon fabrication and optical fiber integration follow the biomimetic design in which a resin (with smaller Young’s modulus) is placed into a mold (divided into two parts). It is also important to mention that the mold also has the supports for the optical fiber positioning as shown in Figure 1. Moreover, the photosensitive single-mode fibers (ThorLabs GF1AA) have three FBGs inscribed via phase mask technique using a nanosecond pulsed laser Nd:YAG centered on 266 nm (LOTIS TII LS-2137ULaser) with 8 ns pulse time, used as described in [30], where each FBG has a physical length of 10 mm. Thus, the integration process involved placing three FBGs in a mold, which was subsequently filled with PU resin. This process yielded a structure reminiscent of a tendon, with the FBGs longitudinally distributed within it.

Polyurethane polymer (PU) was selected as the material for integration with FBG sensors due to its versatility. When combined with rare-earth elements to form nanocomposites, it acquires distinct properties that find extensive applications in industry [31]. Notably, PU is also utilized in medical applications, including implants [32]. To evaluate the mechanical characteristics of the artificial tendon composed of PU and optical fibers, stress–strain tests were performed to determine its elastic modulus (E). Following the guidelines of the ASTM D638 standard, five specimens were subjected to testing using the universal testing machine (MBioPDI, São Carlos, Brazil). It is important to mention that the PU is applied due to its flexibility and aforementioned use in medical devices, which is higher than the one of conventionally used polymer coatings in optical fibers. In addition, the PU is a biocompatible material widely used in many medical applications with developments reported in the last decades [33].

The tendon integrated with FBG sensors undergoes three types of characterization. The first characterization assesses the influence of temperature variation on the Bragg peaks, as indicated by Equation (1). This test is conducted by placing the tendon inside an oven (Ethik Technology, Vargem Grande Paulista, Brazil), where temperature variations between 30 °C and 60 °C are examined in 5 °C increments. At each recorded temperature, the FBG signal is acquired using an optical interrogator si155 (Luna Innovations, Roanoke, VA, USA) at a sampling rate of 10 Hz for 1 min, following a 30 s stabilization period after reaching the desired oven temperature.

Single-axis strain characterizations are conducted on the FBGs before and after their embedding in the tendon to assess the influence of integration process on sensitivity of the sensors. In bare FBG characterization, the ends of the optical fibers are fixed in linear stages with a precision of 10 μm. The initial lengths between the fixed parts of each fiber are measured to calculate the dimensionless strain imposed on the fibers. The dimensionless strain is obtained by dividing the imposed strain by the initial length of the fiber segment. For strain characterization, the signal acquisition is performed using the optical interrogator at a sampling rate of 500 Hz for 30 s. The strain range tested varies from 0 μm to 100 μm in increments of 10 μm. The procedure for strain characterization is repeated for the tendon after the FBGs have been embedded.

**Figure 2:**
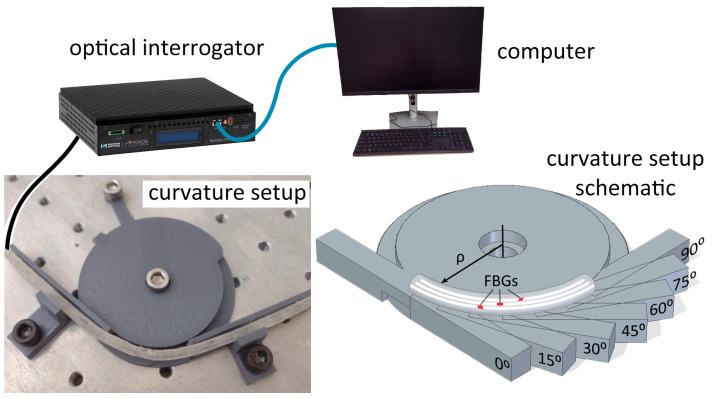
Experimental setup for the FBG-integrated artificial tendon characterization.

In addition to the previous characterizations, a characterization of curvature in the tendon is conducted. This test evaluates how the sensor signal varies when the tendon is subjected to flexion, taking into account the variation in the radius of curvature and the angle of bending. During this test, the entire spectrum emitted by the optical interrogator is captured, allowing for the measurement of wavelengths and signal power. The range of angles tested spans from 0° to 90°, with increments of 15°. Figure 2 shows the experimental setup used on the characterization of the proposed artificial tendon.

In the characterizations, the spectra collected are analyzed using open-source software. The reflected wavelengths are analyzed in the temperature and single-axis strain characterizations. For each measurement, the mean value of the Bragg wavelength ($\lambda_{B}$) is calculated. This data is then used to design a sensor calibration curve, which establishes the correlation between $\lambda_{B}$ and the corresponding temperature or deformation variable to which the sensor is subjected.

In the curvature characterization, the collected signal includes two variables: wavelengths and reflected optical power. The variation in $\lambda_{B}$ is influenced by the deformation, specifically the radius of curvature (ρ). This relationship is derived from the calculation of the bending moment (*M*) in a straight element

(2) $$\frac{1}{\rho }=-\frac{\varepsilon }{y}=\frac{M}{EI}$$

here, *y* represents the distance to the neutral axis, and *I* is the moment of inertia of the cross-sectional area of the element. On the other hand, the reflected optical power is influenced by changes in the angle of tendon curvature. In optical fibers, both micro and macro curvatures can cause power losses.

## 3. Results and Discussion

**Figure 3:**
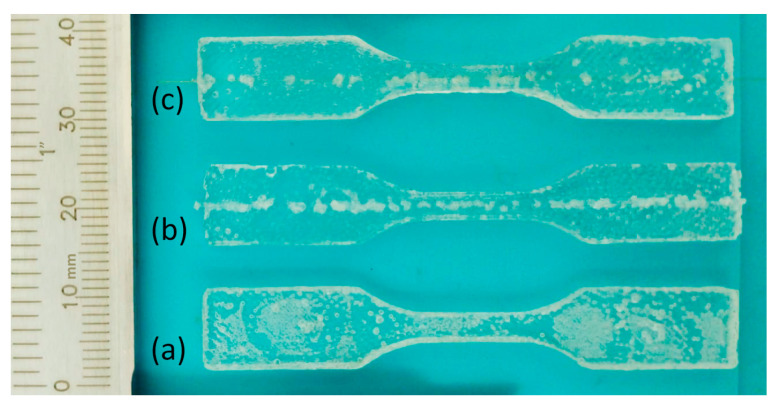
Test specimens. (**a**) PU, (**b**) PU + optical fiber, and (**c**) PU + optical fiber + coating.

In Table 1, the elastic modulus values obtained from the stress–strain tests of the specimens shown in Figure 3 are presented for the PU material, as well as the integration of PU with an SMF-28 optical fiber. The results indicate the influence of fiber integration on the mechanical properties of the composite material. The elastic modulus for the PU material alone is measured to be 4.01 × 10^−2^ GPa. When the PU is integrated with an SMF-28 optical fiber, the modulus of elasticity slightly increases to 3.13 × 10^−1^ GPa. Furthermore, when considering the additional coating layer on the fiber, the modulus of elasticity further increases to 3.93 × 10^−1^ GPa. These values demonstrate the effect of fiber integration on the mechanical characteristics of the PU material, where there is an increase in the Young’s modulus related to the optical fiber, since it has a Young’s modulus of around 70 GPa, which leads to an increase on the artificial tendon Young’s modulus.

**Figure 4:**
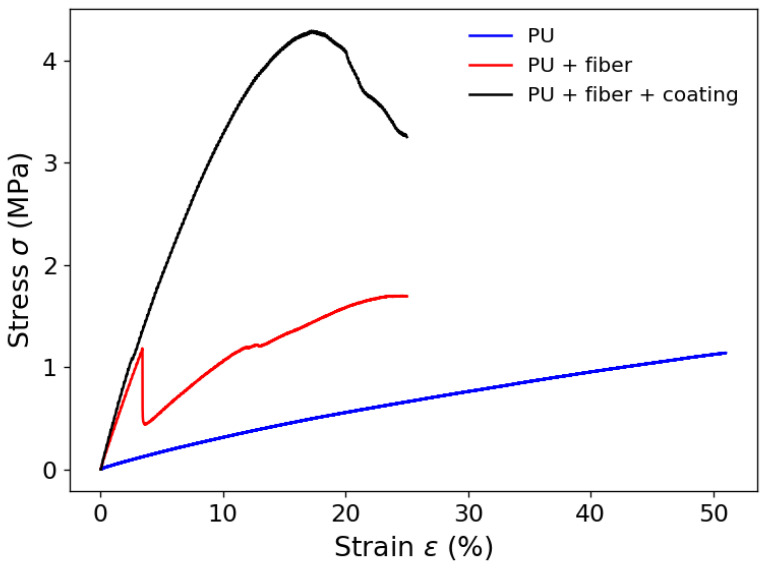
Stress–strain curve of the FBG-integrated artificial tendon.

It is also important to mention that the optical fiber also affects the strain limits on the artificial tendon, where there is a reduction in the strain limit when the optical fiber is integrated in the PU matrix. To that extent, Figure 4 shows the stress–strain curve of the optical-fiber-integrated artificial tendon in which it is possible to observe the region where there is a breakage of the optical fiber, leading to a sharp decrease in the stress response. For this reason, it is possible to infer the influence of the optical fiber on the artificial tendon mechanical properties since there is a 37.45% reduction in the stress when there is the optical fiber breakage in the stress–strain test.

**Figure 5:**
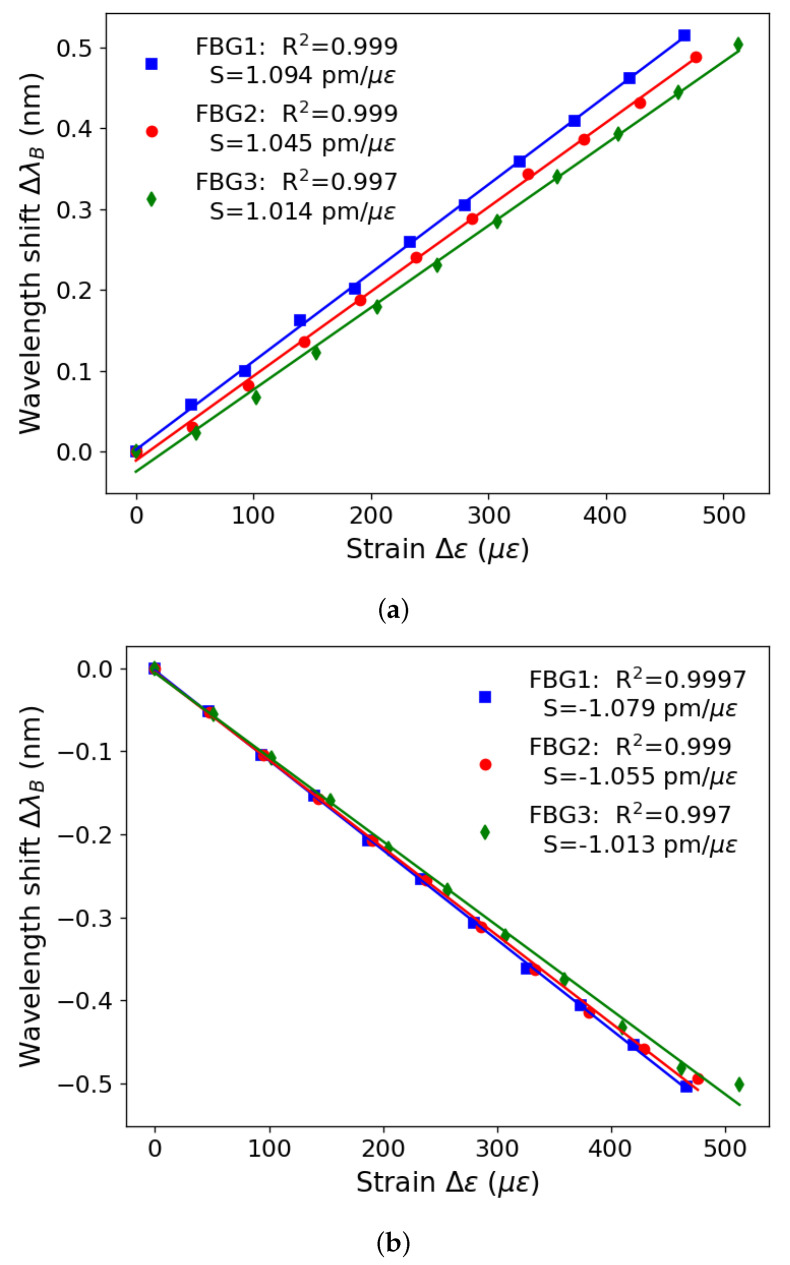
Bare FBGs strain results: (**a**) increasing strain, (**b**) decreasing strain.

Figure 5 presents the results of the strain characterization for three different FBGs. The x-axis represents the strain variation, calculated as the ratio between strain and initial length ($\Delta \varepsilon /L_{0}$). Each measurement has a Δε step of 0.01 mm, and the measured $L_{0}$ values are 214.4 mm, 210.0 mm, and 195.0 mm for FBG1, FBG2, and FBG3, respectively. The y-axis represents the difference between the average wavelength value obtained in each measurement and the average value obtained in the first measurement. From Equation (1), the estimated relationship between the variables is linear, and then linear regressions were performed and shown by the straight lines in Figure 5. Therefore, the determination coefficient (R^2^) that the FBGs presented was above 0.99, calculated for the advance (left) and return (right) results. Furthermore, the sensitivity (S) of the FBG directly depends on how much the gratings move when the optical fiber is deformed. In this way, as the same fiber material was used for recording the FBGs, the value of S differs only in the order of 10^−2^ pm between the tested ones.

**Figure 6:**
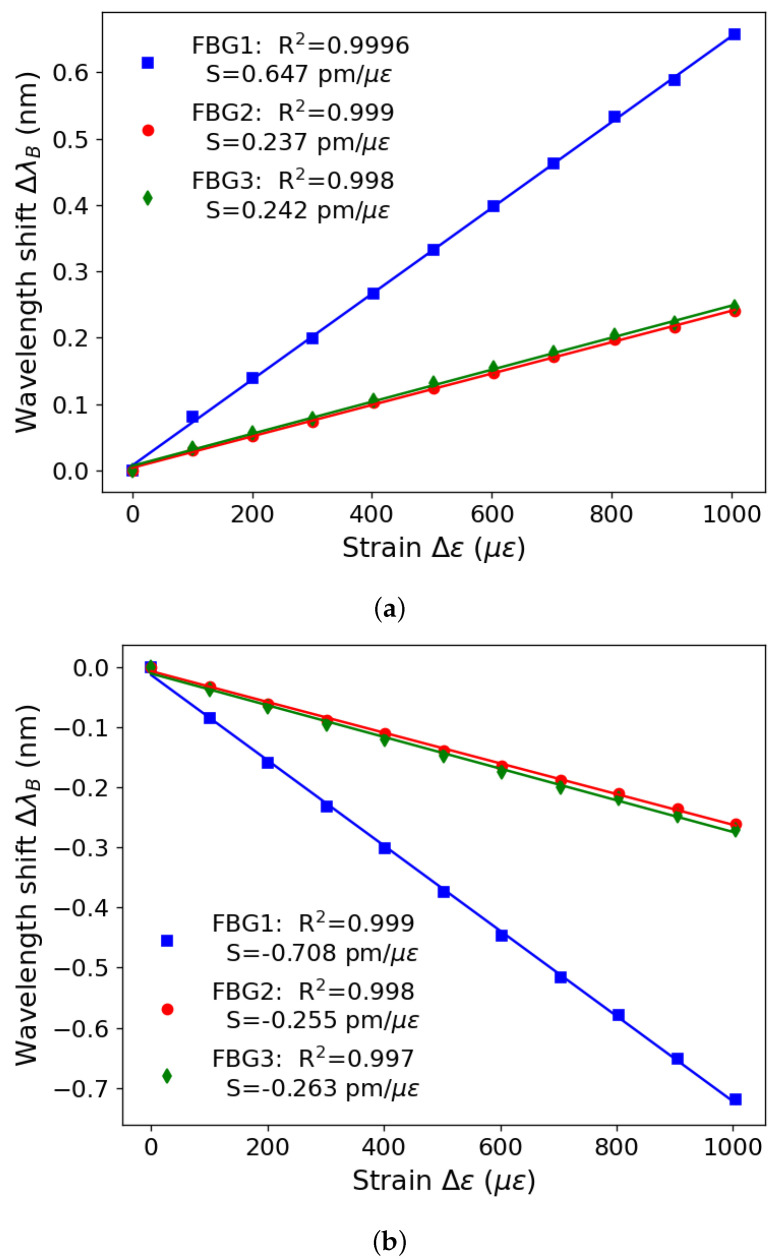
FBG-integrated artificial tendon strain results: (**a**) increasing strain, (**b**) decreasing strain.

Figure 6 presents the results referring to the strain characterization in the FBG-embedded artificial tendon. The same variables represented in Figure 5 are on both axes but with the difference that the $L_{0}$ value was measured at 99.5 mm for the tendon. It can be seen in Figure 6 that FBG1 exhibits an S value of 0.647 pm/με, while FBG2 and FBG3 exhibit an S value closer to 0.24 pm/με. This difference in sensor response is due to the positioning of the FBGs within the tendon. When strain is applied in the longitudinal direction, the material is compressed in the transverse direction. Therefore, the sensors that are closer to the end of the tendon (FBG2 and FBG3) are subjected to a different stress field than the sensor located in the center. This difference in sensitivity can also be seen in [1], in which different positions of the sensors within the structure of a diaphragm result in different responses. The tests were sequentially performed with three cycles for each component and resulted in standard deviations of 36.02 pm, 13.19 pm, and 14.91 pm for FBGs 1, 2, and 3, respectively. Such results indicate a high repeatability of the proposed multifunctional structure.

**Figure 7:**
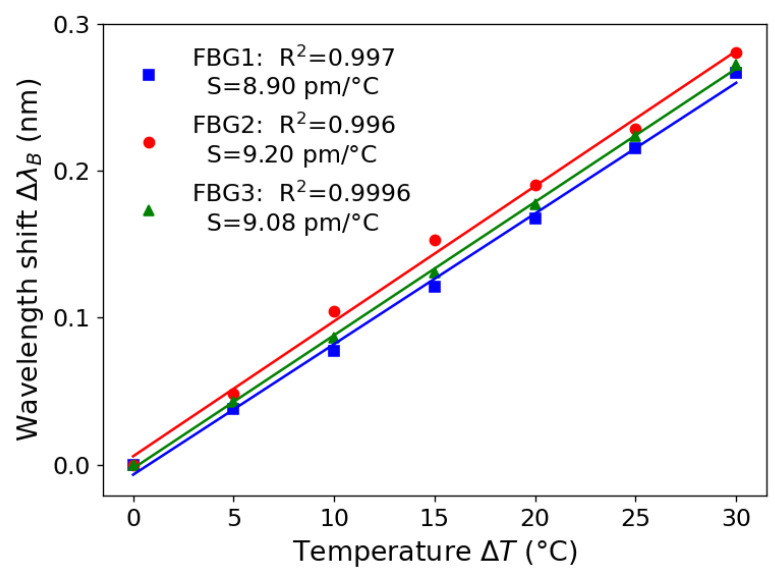
FBG-integrated artificial tendon temperature results.

Figure 7 presents the results of the temperature characterization performed on the tendon. As seen in Equation (1), the relationship between temperature and wavelength shift in FBGs has a linear behavior. It can be observed with the determination coefficients that this relation does not change with the embedding of the sensors in the tendon. It is noteworthy that the sensitivity of FBG sensors to temperature variation depends on the heat conduction of the PU. Therefore, the presence of heterogeneity resulting from the fabrication of the tendon is a factor that influences the temperature profile within the material. For this reason, the position of the sensors within the tendon results in differences between their responses.

**Figure 8:**
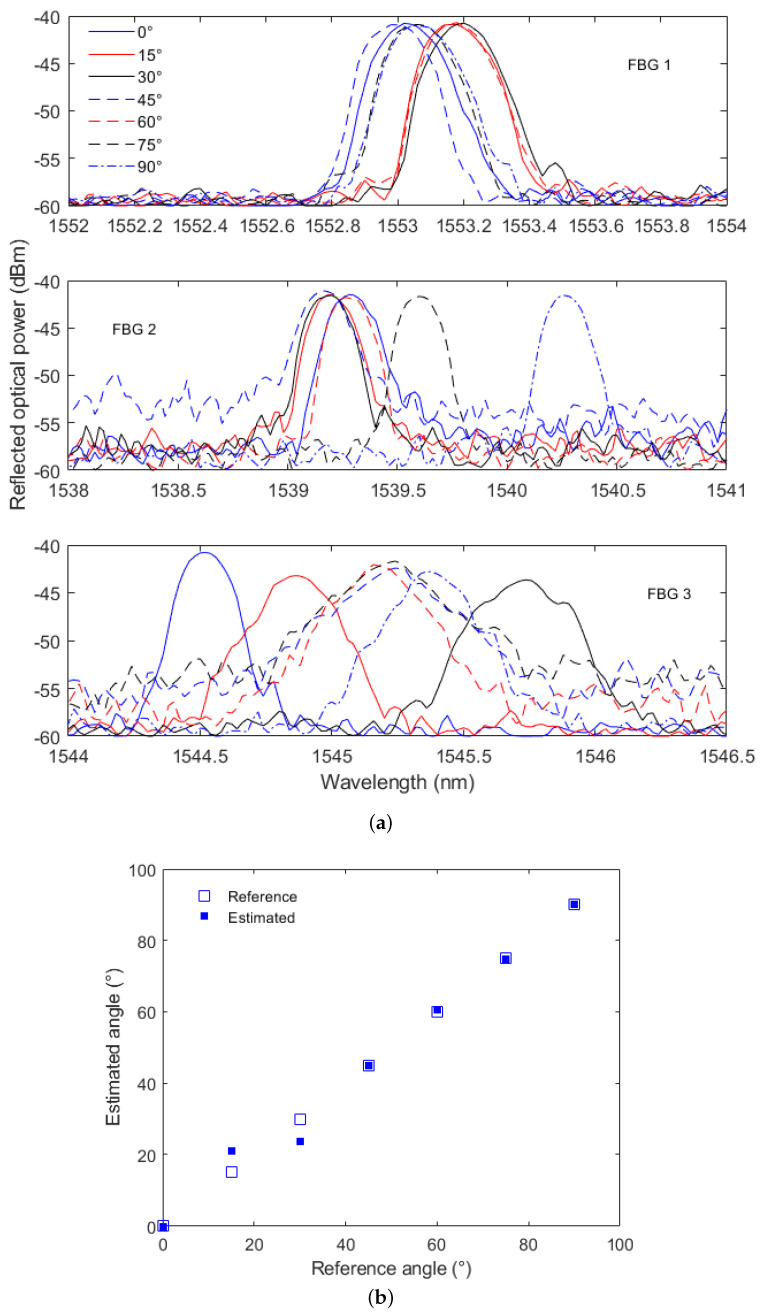
FBG-integrated artificial tendon curvature results: (**a**) wavelength shift, (**b**) estimated angle.

Finally, the last characterization test on the FBG-embedded artificial tendon is presented in Figure 8a, where it is possible to observe the FBGs’ reflected spectra at different angle conditions. To that extent, it is possible to observe the variations in the spectral features as a function of the applied angle on the tendon. In this case, there is a higher variation on the reflected optical power, which is related to the macrocurvatures applied on the optical fibers. It is important to mention that the bending in the fiber (especially on the FBG region) leads to different spectral variation on the sensor response, which is related to the specificity of such mechanical loading. In this case, there is a distributed quadratic strain along the tendon (and the embedded optical fiber) as demonstrated in [34]. Moreover, the optical attenuation induced by the bending in the optical fiber leads to a variation in the reflected optical fiber. For this reason, the wavelength shift and the optical attenuation of all three FBGs are used on a multifeature regression model for the curvature angle estimation.

The curvature angle in this case is estimated from the reflected optical power and the wavelength shift as a function of the angle, where their variations for each FBG can be observed in Figure 8a. Thus, the wavelength shift and the optical power variation are used in a polynomial relation to obtain the curvature angle, where such a relation can be used for each FBG. Then, all three estimated angles are combined into a single angle estimation using a linear relation as shown in Equations (3) and (4) for the angle estimation of each FBG and angle estimation combination, respectively. In addition, Figure 8b shows the values of the estimated and reference angles considering all three FBGs of the proposed FBG-embedded artificial tendon, where both optical power variation and the wavelength shift are used in a polynomial regression. From the data of reflected optical power and wavelength shift of the embedded FBGs, it is possible to infer the bending angle and its direction from the spectral feature variations with a root mean squared error of around 3.25°. In addition, the strain distribution along the tendon can be estimated from the strain results of each FBG embedded in the proposed artificial tendon.

(3) $$\alpha_{1-3}=a_{1-3}\times \Delta \lambda_{B}{}_{1-3}+b_{1-3}\times \Delta P_{1-3}+c_{1-3}\times \Delta \lambda_{B}{}_{1-3}^{2}+d_{1-3}$$

(4) $$\alpha_{est}=x_{1}\times \alpha_{1}+x_{2}\times \alpha_{2}+x_{3}\times \alpha_{3}+x_{4}$$

## 4. Conclusions

This paper presented the development of an artificial tendon with embedded optical fibers. In this case, three optical fibers, with one FBG each, are embedded in the PU matrix to obtain a biomimetic design of the tendon. The mechanical properties of the proposed artificial tendon were characterized via stress–strain analysis, where it is possible to observe an eight-fold increase of the Young’s modulus when the optical fibers are embedded into the PU matrix. In addition, there is a reduction in the strain limits on the FBG-embedded artificial tendon since there is a breakage of the optical fiber when the strain is close to 10%. Considering the artificial tendon characterization as a function of the strain, a reduction in the axial strain sensitivity related to the FBG positioning in the tendon was observed for all three FBGs. In this case, there is a 41%, 77%, and 76% reduction in the strain sensitivity for the FBG1, FBG2, and FBG3, respectively. In contrast, when the temperature response is analyzed, there are similar sensitivities of FBGs with minor variations related to the heterogeneity on the PU matrix that can lead to variations on the heat transfer to the FBGs. Finally, the bending characterizations indicated the sensitivities of the FBGs’ spectral features, especially on the wavelength shift and the reflected optical fiber, which can be combined to obtain the directional strain distribution as well as the plane shape reconstruction of the proposed artificial tendon. Therefore, the proposed approach resulted in a biomimetic multifunctional tendon, which can be used not only in tendon-driven soft actuators but also in biomechanical devices for simultaneous assessment of different parameters in a device that not only has customized structural properties but also has self-sensing capabilities. Future works include the implementation of the device in tendon driven actuators. In addition, the development of new distributed sensing approaches are envisaged for the 3D real-time shape reconstruction of the tendon’s complex structure.

## Figures and Tables

**Table 1:** Mechanical properties obtained from the stress–strain test.

Material	E (GPa)
PU	4.01 × 10^−2^
PU + optical fiber	3.13 × 10^−1^
PU + optical fiber + coating	3.93 × 10^−1^

## Data Availability

The data presented in this study are available on request from the corresponding author.
